# Associations Between Self-Reported Sleep Quality, Fatigue Severity, Factors Associated With Successful Cessation, and Cessation Beliefs Among Regular Smokers

**DOI:** 10.1093/ntr/ntad231

**Published:** 2023-11-23

**Authors:** Joe A Matthews, Hannah M Sallis, Maddy L Dyer, Ryan McConville, Hanna Isotalus, Angela S Attwood

**Affiliations:** School of Psychological Science, University of Bristol, Bristol, UK; Department of Electrical and Electronic Engineering, University of Bristol, Bristol, UK; Medical Research Council Integrative Epidemiology Unit, University of Bristol, Bristol, UK; Integrative Cancer Epidemiology Programme at the University of Bristol, Bristol, UK; Medical Research Council Integrative Epidemiology Unit, University of Bristol, Bristol, UK; CAMH (Centre for Academic Mental Health), Population Health Sciences, Bristol Medical School, University of Bristol, Bristol, UK; School of Psychological Science, University of Bristol, Bristol, UK; Medical Research Council Integrative Epidemiology Unit, University of Bristol, Bristol, UK; Department Engineering and Mathematics, Ada Lovelace Building, University of Bristol, University Walk, Bristol, UK; Department of Electrical and Electronic Engineering, University of Bristol, Bristol, UK; School of Psychological Science, University of Bristol, Bristol, UK; Medical Research Council Integrative Epidemiology Unit, University of Bristol, Bristol, UK; Integrative Cancer Epidemiology Programme at the University of Bristol, Bristol, UK; Bristol Biomedical Research Centre at the University of Bristol, Bristol, UK

## Abstract

**Background:**

Smokers report poorer sleep than nonsmokers and sleep quality deteriorates further during cessation, increasing risk of smoking relapse. Better understanding of the relationship between sleep and relapse-related outcomes could inform novel approaches to smoking cessation support. The aim of this study was to investigate same-day associations of self-reported sleep quality and fatigue severity with factors associated with successful cessation and cessation beliefs, among regular smokers.

**Methods:**

This cross-sectional observational study (*n* = 412) collected self-reported sleep quality, fatigue severity, and factors associated with successful cessation and cessation beliefs among regular smokers via an online survey (60% male).

**Results:**

There was evidence of an association between sleep quality (SQ) and reduced 24-hour (β = −0.12, *p =* .05) and lifetime (β = −0.09, *p =* .04) abstinence self-efficacy. In addition, poorer SQ and higher fatigue severity (FS) were associated with increased smoking urges (SQ: β *=* 0.27, *p* < .001; FS: β *=* 0.32, *p <* .001), increased barriers to cessation (SQ: β *=* 0.19, *p* < .001; FS: β *=* 0.32, *p* < .001), and increased perceived risks to cessation (SQ: β *=* 0.18, *p* < .001; FS: β *=* 0.26, *p* < .001). Fatigue severity was weakly associated with increased perceived benefits to cessation (β *=* 0.12, *p* = .017).

**Conclusions:**

Self-reported sleep quality and fatigue severity were associated with multiple factors associated with successful cessation and cessation beliefs. Further research is needed to extend these findings by using different methods to identify the temporal direction of associations and causality.

**Implications:**

This study is the first to examine associations between sleep quality, fatigue severity, and factors associated with successful cessation and cessation beliefs. Findings show that both sleep quality and fatigue severity are associated with multiple factors associated with successful cessation and could be modifiable targets for future smoking cessation interventions. Furthermore, our data suggest that fatigue severity has an independent effect on multiple factors associated with successful cessation when accounting for sleep quality. This indicates that fatigue, independent of sleep quality, could be an important factor in a quit attempt.

## Introduction

Smoking is one of the leading causes of mortality, morbidity, and disability.^1^ Each year, smoking is responsible for 8 million deaths globally^2^ and approximately 150 million disability-adjusted life-years lost.^3^ Despite 55% of UK smokers reporting a desire to quit smoking^4^ and approximately one-third engaging in a quit attempt each year, only 5% of smokers are successful.^5^ This is problematic for many governments who have ambitions to create smoke-free societies (adult smoking prevalence of 5% or less), such as New Zealand (by 2025), England and Finland (by 2030),^6^ Scotland (by 2034), and Germany (by 2040).^7,8^ It is therefore important to understand the factors influencing current smoking behavior and the mechanisms contributing to a successful quit attempt. Despite multiple effective pharmacological and behavioral interventions targeting smoking cessation,^9^ smoking prevalence is still high. A growing area of research is to target wider lifestyle behaviors that are known to impact smoking behavior and/or cessation, one of which is sleep.

Components of sleep quality such as sleep onset latency, sleep efficiency, sleep disturbances, and sleep duration are potentially linked with poor cessation outcomes.^10^ Several studies report that poor sleep quality before a quit attempt increases the risk of smoking relapse.^11–15^ Furthermore, previous research reports associations between poor self-reported sleep quality and smoking relapse risk factors, such as decreased quit-day abstinence self-efficacy,^16^ increased smoking urges,^17^ and increased delay discounting.^18^ However, further research is needed to identify potential underlying mechanisms underpinning such relationships, as sleep is a potential modifiable target for novel smoking cessation interventions.

One potential influential factor is fatigue, which reflects the experience of tiredness, lacking energy, and feeling exhausted.^19^ Previous research has found that sleep quality and fatigue are associated in multiple populations including in people with pain,^20^ fibromyalgia,^21^ and cancer.^22^ However, although fatigue is associated with and can be predicted by poor sleep quality in a variety of conditions, previous research suggests that fatigue and poor sleep quality are separate constructs.^23^ Fatigue as a construct is multidimensional and is not necessarily linked with daytime sleepiness.^24^ Fatigue may decrease the likelihood of smoking cessation by affecting known relapse risk factors such as abstinence self-efficacy, mood, and smoking urges, or by decreasing the perceived value of future rewards or increasing perceived barriers or risks regarding cessation. While previous work has explored the role of fatigue in substance use maintenance^25,26^ and relapse,^27^ to the best of our knowledge, no studies have explored associations between fatigue severity and factors associated with successful cessation or perceived cessation barriers. Furthermore, the potential effect of fatigue on cessation could be exacerbated not only by poor sleep, but by nicotine withdrawal during cessation, as stimulation from nicotine attenuates fatigue.^28^

The current study investigated associations between self-reported sleep quality and fatigue severity (exposures) and multiple known factors associated with successful cessation and cessation beliefs (outcomes) among regular smokers. Factors associated with successful smoking cessation were included if they were known to impact cessation and could theoretically be linked with sleep quality and/or fatigue. As discussed previously, abstinence self-efficacy and smoking urges are strong predictors of smoking cessation outcomes^29^ and known to be impacted by sleep.^16,17^ Similarly, delay discounting predicts future smoking, and in particular it decreases the likelihood of successful smoking cessation.^30^ Furthermore, individuals with poorer habitual sleep quality discount rewards more heavily than those with better habitual sleep.^18^ We hypothesized that poorer sleep quality and greater fatigue severity would each be associated with greater delay discounting, higher smoking urges, lower abstinence self-efficacy, lower perceived benefits of cessation, lower readiness to quit, greater perceived risks of cessation, and greater perceived barriers to cessation. The secondary aim was to identify whether there is an independent association between fatigue severity and factors associated with successful cessation after accounting for sleep quality and theoretically relevant covariates. This will provide insight on whether future models examining associations between sleep quality and factors associated with successful cessation should include fatigue severity.

## Methods

### Design

This was a cross-sectional observational study (online survey) investigating associations between self-reported sleep quality and fatigue severity (exposures) and factors associated with successful cessation (24-hour and lifetime abstinence self-efficacy, readiness to quit, smoking urges, and delay discounting) and cessation beliefs (barriers to cessation, perceived risks and benefits to cessation) (outcomes). Ethics approval was obtained from the School of Psychological Science Research Ethics Committee at the University of Bristol (reference: 117349). The study protocol was preregistered on the Open Science Framework (https://osf.io/h784b).

### Participants

We recruited 441 participants through the Prolific crowd-sourcing platform (www.prolific.co). Participants were eligible if they were 18 years of age or over, had English as a first language or similar level of fluency, and were a regular smoker (defined as smoking at least 5 cigarettes per day, for at least 3 months).

While interested in both sleep quality and fatigue severity, this study was powered based on fatigue severity as the explanatory variable as this was our primary association of interest. We performed a sample size calculation using GPower 3.1 for a fixed model linear regression, with a power of 0.95 and an alpha level of 0.01. An Δ*R*^2^ value of 0.07 (explanatory variable, fatigue severity; outcome variable, barriers to cessation) and a residual variance of 0.68 were identified based on the smallest effect size from a previous study of fatigue severity and cessation beliefs.^21^ This gave a total sample size estimate of 408 participants. To account for possible post hoc removal of outliers and attention check failures, we recruited 440 participants.

### Procedure

Prolific members who met the eligibility criteria received a study invitation via their online account, which included a link to the survey hosted on the Qualtrics platform (https://www.qualtrics.com/). Participants were first shown an information statement explaining the study and inviting them to contact the research team if they had any questions. Participants provided informed consent electronically. They then completed the survey questions (see Measures section). Halfway through the survey participants received an attention check. After completing the survey, participants were shown a debrief statement on the final webpage. The survey took approximately 20 minutes to complete, and participants were reimbursed £2.50 via their Prolific account.

### Measures

#### Demographics Variables and Smoking Characteristics

Age, sex, country of residence, and education (“higher education or professional/vocational equivalents,” “A-levels or vocational level 3 or equivalents,” “GCSE/O-Level grade A*‐C or vocational level 2 or equivalents,” “qualifications at level 1 and below,” “other qualifications: level unknown,” or “no qualifications”), nicotine dependence (Fagerström Test of Nicotine Dependence [FTND]^31^), electronic cigarette user status, 12-month quit attempt history, and cigarettes smoked per day were used to describe the sample. Age, sex, education, and nicotine dependence were included as covariates in regression analyses and were selected based on their a priori relevance and/or their associations with smoking behavior, sleep quality, and fatigue severity in the literature (ie, their potential to be a confounder).

#### Exposure Variables

##### Sleep Quality.

The Pittsburgh Sleep Quality Index (PSQI)^32^ has been widely used in the context of sleep and behavioral research. Nineteen items generate a global sleep quality score. Validity and reliability of this instrument are reported elsewhere.^32,33^ Global sleep quality scores range from 0 to 21, with higher scores indicating worse sleep quality.

##### Fatigue Severity.

We used the Fatigue Severity Scale (FSS), a well-validated nine-item scale of fatigue severity.^34^ Questionnaire items are rated on a 7-point scale from 1 “no impairment” to 7 “severe impairment.” Total scores range from 9 to 63, with higher scores indicating greater chronic fatigue severity.

### Outcome Variables

#### Factors Associated With Successful Smoking Cessation

##### Readiness to Quit.

Participants completed the Readiness to Quit Ladder,^35^ which is single-item measure of motivation to change smoking behavior. It uses a 10-point ordinal scale with responses ranging from 1 “I have decided to continue smoking” to 10 “I have already quit smoking.” This instrument performs well when predicting smoking rate, quit attempts, and cessation, and is associated with cognitive and behavioral indicators of readiness to consider smoking abstinence.^35,36^

##### Abstinence Self-Efficacy.

This was measured by two items, which were analyzed separately: “If you were to quit smoking today, how confident are you that you would not smoke within the next 24 hours” (24-hour abstinence self-efficacy) and “If you were to quit smoking today, how confident are you that you would remain abstinent?” (lifetime abstinence self-efficacy). Responses were measured on a 100-point visual analog scale from 0 “not confident” to 100 “very confident.”

##### Smoking Urges.

Participants completed the Questionnaire of Smoking Urges-Brief (QSU-Brief)^37^; a 10-item questionnaire using 7-point scale from 1 “strongly disagree” to 7 “strongly agree.” This measures overall smoking urges with total scores ranging from 10 to 70, with higher scores indicating a greater urge to smoke.

##### Delay Discounting.

The Kirby Monetary Choice Questionnaire (MCQ) is a widely used 27-item measure of delay discounting.^38^ Participants were presented with a hypothetical choice between two sums of money, one sum immediately and a larger sum after a varying amount of time. For example, participants could be asked to answer if they would prefer £10 immediately or £15 in 1 month.

#### Cessation Beliefs

##### Barriers to Cessation.

The Barriers to Smoking Cessation Scale (BCS)^39^ comprises of 19 items each using 4-point scales from 0 “not a barrier” to 4 “large barrier,” and describes general circumstances or specific perceived barriers that may interfere with a quit attempt. It has been shown to be a reliable measure of perceived barriers and is related to several affective and smoking processes that may interfere with smoking cessation.^40^ Total scores range from 0 to 76, with higher scores indicating greater perceived barriers to cessation.

##### Perceived Risks and Benefits of Cessation.

The Perceived Risks and Benefits Questionnaire (PRBQ)^41^ is a 40-item questionnaire assessing perceived risks (eg, weight gain) and benefits (eg, social approval) of smoking cessation. Each item is assessed on a 7-point scale from 1 “no chance” to 7 “certain to happen.” Total scores for both risks and benefits range from 20 to 140, and the higher the score, the greater the perceived benefits or risks to cessation. This instrument has demonstrated superior reliability and validity when compared to other self-reported measures of personal health risk.^41^

#### Attention Check

One attention check question was embedded within the survey (“When was the last time you flew to Mars?”) with response options “never,” “a few days ago,” “weeks ago,” and “months ago.” Only “never” responses were considered satisfactory to pass the attention check.

### Data Analysis

Analyses were conducted using SPSS Statistics Version 26.^42^ Prior to analysis, data from participants who failed the attention check were removed and data were checked for violation of assumptions for linearity, independence of residuals, homoscedasticity, and multicollinearity.

#### Primary analyses

We performed separate linear regression models with factors associated with successful cessation and cessation beliefs (abstinence self-efficacy, smoking urges, delay discounting, readiness to quit, barriers to cessation, and perceived risks and benefits of cessation) as the outcome variables and sleep quality and fatigue severity as the exposure variables. As stated in the protocol, we planned to collect and analyze acute sleep quality but these data were not collected due to technical error. We report unadjusted results and results adjusted for demographic variables and nicotine dependence. Before running the delay discounting regression models, the MCQ Automated Scorer^43^ was used to transform delay discounting data from the MCQ to discounting rate for each participant. Discounting rate was then used as the outcome variable in the regression model.

#### Secondary analyses

To evaluate if there was an independent association between fatigue severity and smoking-related outcomes after accounting for sleep quality and adjusting for covariates, hierarchical regression analyses were conducted for outcome variables that displayed evidence of associations for sleep quality and fatigue severity in previous linear regression analyses. Covariates were entered during step 1 of each model (sex, age, education, and nicotine dependence). Sleep quality was entered during step 2 and fatigue severity was entered during step 3 of each model. *R*^2^, Δ*R*^2^, and standardized (*b*) and unstandardized regression point estimates (β) are reported.

## Results

After excluding individuals whose self-reported level of smoking did not meet our definition of a “regular smoker” (*n* = 16) and those who failed the attention check question (*n* = 13), our sample consisted of 412 participants (Table 1). Participants were mostly male (60%), aged between 18 and 74 years (*M* = 35, *SD* = 13), and reported smoking between 5 and 72 cigarettes per day (*M* = 13, *SD* = 7). Almost half the sample (45%) had engaged in a quit attempt within 12 months of the survey and showed low to moderate levels of nicotine dependence, scoring between 0 and 7 on the FTND (*M* = 3.7, *SD* = 1.9).

**Table 1.** Participant (*n* = 412) Demographic Variables, Sleep, and Smoking Characteristics

Demographic variables	
Sex (male), *n* (%)	248 (60)
Age (years), mean, (*SD*)	34.2 (12.7)
	*N* (%)
Country of residence	
United Kingdom	156 (37.6)
Poland	65 (15.8)
United States	38 (9.2)
Portugal	34 (8.3)
Italy	33 (8.0)
Greece	17 (4.1)
Spain	15 (3.6)
South Africa	8 (1.9)
Other	46 (11.2)
Education	
Higher education	204 (49.5)
A-levels or equivalent or ≥ level 3	74 (18.0)
GCSE or equivalent A*-C or ≥ level 2	78 (18.9)
Qualifications ≤ level 1	20 (4.9)
Other qualifications	25 (6.1)
No qualifications	11 (2.6)
Smoking characteristics	
Cigarettes per day, mean (*SD*)	13 (7)
Nicotine dependence, mean (*SD*)	2.5 (1.4)
Quit attempt within 12 months of survey, *n* (%)	184 (44.67)
Comorbid use of vape or e-cigarette, *n* (%)	154 (37.4)
Sleep and fatigue variables	
Global sleep quality, mean (*SD*)	6.16 (3.19)
Fatigue severity, mean (*SD*)	37.80 (11.72)

Nicotine dependence measured by the FTND, global sleep quality measured by the PSQI, and fatigue severity measured by the FSS. FSS = Fatigue Severity Scale; FTND = Fagerström Test of Nicotine Dependence; PSQI = Pittsburgh Sleep Quality Index.

### Primary analyses

Poorer sleep quality (SQ) and greater fatigue severity (FS) were associated with reduced 24-hour abstinence self-efficacy (SQ: *b = −*2.09, 95% CI *−*3.04, *−*1.13, *p <* .001; FS: *b* = *−*0.33, 95% CI *−*0.60, *−*0.07, *p =* .013), reduced lifetime abstinence self-efficacy (SQ: *b = −*1.19, 95% CI *−*1.93, *−*0.45, *p =* .002; FS: *b = −*0.29, 95% CI *−*0.50, *−*0.09, *p =* .004), increased smoking urges (SQ: *b =* 1.45, 95% CI 1.01, 1.89, *p* < .001; FS: *b =* 0.47, 95% CI 0.36, 0.59, *p <* .001), increased barriers to cessation (SQ: *b =* 0.80, 95% CI 0.51, 1.09, *p* < .001; FS: *b =* 0.32, 95% CI 0.25, 0.40, *p* < .001), and increased perceived risks to cessation (SQ: *b =* 0.08, 95% CI 0.05, 0.11, *p* < .001; FS: *b =* 0.03, 95% CI 0.02, 0.04, *p* < .001). Fatigue severity was also associated with an increase in perceived benefits to cessation (*b =* 0.01, 95% CI 0.01, 0.01, *p* = .002). There was no clear evidence of an association between sleep quality or fatigue severity with delay discounting or readiness to quit.

When adjusting for demographic variables and nicotine dependence, fatigue severity was no longer associated with neither 24-hour abstinence self-efficacy (*b = −*0.10, 95% CI *−*0.34, 0.13, *p* = .392) nor lifetime abstinence efficacy (*b = −*0.17, 95% CI *−*0.37, 0.02, *p* = .082). All other associations remained after adjusting for the covariates, but effect estimates were attenuated (Table 2).

**Table 2.** Cross-Sectional Associations of Sleep Quality and Fatigue Severity With Smoking Relapse Risk Factors and Cessation Beliefs

		Sleep quality				Fatigue Severity			
		*b* (95% CI)	β	*R* ^2^	*p* value	*b* (95% CI)	β	*R* ^2^	*p* value
24-hour abstinence self-efficacy	Unadjusted	*−*2.09 (*−*3.04, *−*1.13)	*−*0.21	0.04	<.001	*−*0.33 (*−*0.60, *−*0.07)	*−*0.12	0.01	.013
	Adjusted	*−*1.22 (*−*2.06, *−*0.37)	*−*0.12	0.29	.005	*−*0.10 (*−*0.34, 0.13)	*−*0.04	0.28	.392
Lifetime abstinence self-efficacy	Unadjusted	*−*1.19 (*−*1.93, *−*0.45)	*−*0.16	0.02	.002	*−*0.29 (*−*0.50, *−*0.09)	*−*0.14	0.01	.004
	Adjusted	*−*0.71 (*−*1.41, *−*0.01)	*−*0.09	0.16	.048	*−*0.17 (*−*0.37, 0.02)	*−*0.08	0.15	.082
Smoking urges	Unadjusted	1.45 (1.01, 1.89)	0.31	0.09	<.001	0.47 (0.36, 0.59)	0.37	0.13	<.001
	Adjusted	1.25 (0.82, 1.68)	0.27	0.15	<.001	0.41 (0.30, 0.53)	0.32	0.18	<.001
Delay discounting	Unadjusted	0.00 (0.00, 0.00)	0.09	0.01	.081	0.00 (*−*0.00, 0.00)	*−*0.03	0.00	.533
	Adjusted	0.00 (0.00, 0.01)	0.09	0.01	.083	0.00 (*−*0.00, 0.00)	*−*0.02	0.01	.699
Barriers to cessation	Unadjusted	0.80 (0.51, 1.09)	0.26	0.06	<.001	0.32 (0.25, 0.40)	0.38	0.15	<.001
	Adjusted	0.59 (0.31, 0.87)	0.19	0.15	<.001	0.27 (0.19, 0.35)	0.32	0.21	<.001
Risks of cessation	Unadjusted	0.08 (0.05, 0.11)	0.25	0.06	<.001	0.03 (0.02, 0.04)	0.33	0.11	<.001
	Adjusted	0.06 (0.03, 0.08)	0.18	0.22	<.001	0.02 (0.02, 0.03)	0.26	0.25	<.001
Benefits to cessation	Unadjusted	0.01 (*−*0.01, 0.03)	0.05	0.00	.287	0.01 (0.00, 0.01)	0.15	0.02	.002
	Adjusted	0.00 (*−*0.01, 0.03)	*−*0.02	0.01	.682	0.01 (0.00, 0.01)	0.12	0.02	.017
Readiness to quit	Unadjusted	0.02 (*−*0.07, 0.03)	*−*0.04	0.00	.405	*−*0.01 (*−*0.02, 0.00)	*−*0.10	0.00	.264
	Adjusted	*−*0.03 (*−*0.09, 0.02)	*−*0.07	0.00	.201	*−*0.01 (*−*0.03, 0.00)	*−*0.0	0.01	.096

*N* = 412. *b* = unstandardized coefficient; CI = confidence interval; β = standardized coefficient. Adjusted: adjusted for age, sex, education, and nicotine dependence.

### Secondary analyses

After accounting for covariates and sleep quality, there was still evidence of an association between fatigue severity and smoking urges, perceived barriers to cessation, perceived risks of cessation, and perceived benefits of cessation. Furthermore, the inclusion of fatigue in the model attenuated the association between sleep quality and smoking urges (*b =* 0.73, 95% CI 0.27, 1.20, *p = .*002) and there was no longer an independent association between sleep quality and both perceived barriers to cessation (*b =* 0.21, 95% CI *−*0.10, 0.51, *p* = .183) and perceived risks to cessation (*b =* 0.02, 95% CI *−*0.01, 0.01, *p* = .682) (Table 3).

**Table 3.** Hierarchical Regression Results of Models Including Theoretically Relevant Covariates, Sleep Quality, and Fatigue Severity With Smoking Relapse Risk Factors and Cessation Beliefs

Model 1: 24-hour abstinence self-efficacy				
Variable	Step 2		Step 3	
	*b*	β	*b*	β
SQ	*−*1.22*	*−*0.12	*−*1.31*	*−*0.13
FS			0.06	0.02
*R* ^2^ (adjusted *R*^2^)	0.298 (0.289)		0.298 (0.288)	
Δ*R*^2^	0.014		0.000	
Model 2: Lifetime abstinence self-efficacy				
	Step 2		Step 3	
Variable	*b*	β	b	β
SQ	*−*0.71*	*−*0.09	*−*0.54	*−*0.07
FS			*−*0.11	*−*0.05
*R* ^2^ (adjusted *R*^2^)	0.166 (0.155)		0.168 (0.155)	
Δ*R*^2^	0.008		0.002	
Model 3: Smoking urges				
	Step 2		Step 3	
Variable	b	β	b	β
SQ	1.25**	0.15	0.73*	0.16
FS			0.33**	0.25
*R* ^2^ (adjusted *R*^2^)	0.163 (0.153)		0.209 (0.198)	
Δ*R*^2^	0.067		0.046	
Model 4: Barriers to cessation				
	Step 2		Step 3	
Variable	b	β	b	β
SQ	0.59**	0.19	0.21	0.07
FS			0.24**	0.29
*R* ^2^ (adjusted *R*^2^)	0.163 (0.153)		0.225 (0.213)	
Δ*R*^2^	0.035		0.062	
Model 5: Risks of cessation				
	Step 2		Step 3	
Variable	b	β	b	β
SQ	0.06**	0.18	0.02	0.08
FS			0.02**	0.23
*R* ^2^ (adjusted *R*^2^)	0.226 (0.217)		0.264 (0.253)	
Δ*R*^2^	0.029		0.038	
Model 6: Benefits of cessation				
	Step 2		Step 3	
Variable	b	β	b	β
SQ	0.00	0.02	*−*0.00	*−*0.04
FS			0.01*	0.14
*R* ^2^ (adjusted *R*^2^)	0.023 (0.011)		0.264 (0.023)	
Δ*R*^2^	0.001		0.014	

*N* = 412. SQ = sleep quality; FS = fatigue severity; Step 1 = covariates; Step 2 = sleep quality adjusted for covariates; Step 3 = fatigue severity adjusted for sleep quality and covariates; *b* = unstandardized coefficient; β = standardized coefficient; *p < .05, **p < .01; covariates = age, sex, education, and nicotine dependence.

## Discussion

This is the first study to examine associations between both sleep quality and fatigue severity, and factors associated with cessation and cessation beliefs. As hypothesized, we observed evidence of an association between both poorer sleep quality and higher fatigue severity with multiple factors associated with cessation and cessation beliefs. Associations remained for the majority of outcome variables after adjusting for covariates. Results from our secondary analyses indicated that inclusion of fatigue in the models attenuated the associations between sleep quality and smoking urges, barriers to cessation, and perceived risks to cessation.

Few studies have investigated associations between sleep quality and factors associated with successful smoking cessation in smokers. Two studies have examined the relationship between sleep quality and smoking-related relapse risk factors in smokers^17^ and young smokers^44^ using cross-sectional study designs. While our findings are not directly comparable to the study by Dugas and colleagues^44^ because of a different selection of smoking cessation variables (craving and withdrawal), they also found evidence of associations between sleep quality and multiple smoking cessation factors. Our study supports findings by Purani and colleagues^17^ that poorer sleep quality is associated with increased smoking urges.

Other research has found no association between general sleep quality and urge to smoke; however, this may be explained by differences in study designs. Urge to smoke was an exposure variable and researchers investigated its impact on sleep quality.^45^ Participants only filled out the questionnaire shortly after their last cigarette of the day which likely lowered the urge to smoke.^45^ We also found evidence that sleep quality was associated with both 24-hour and lifetime abstinence self-efficacy, supporting previous findings that smokers who had poor sleep quality at baseline had significantly lower abstinence self-efficacy on their quit day.^16^ It has been hypothesized that difficulty with both sleep onset and disturbance may lead to a perception of uncontrollability and a reduction in self-efficacy to make lifestyle changes such as quitting smoking.^16^ Examining the role of such mediating mechanisms was outside the scope of this study, but future work should consider longitudinal and experimental methods to determine the nature of such relationships and causality.

In the general population, research has found individuals with poorer habitual sleep quality discount rewards more heavily than those with better habitual sleep quality.^18^ However, our study found no robust evidence of an association in smokers. As smokers are more likely to exhibit steeper discounting rates than nonsmokers,^46^ we explored the possibility of a ceiling effect within our data. However, visual inspection of the data did not support this and our selected measure of delay discounting has displayed a relative absence of problems with floor and ceiling effects.^47^ Previous work has suggested that a binary choice task such as the MCQ requires participants to make several choices which is time-consuming, and when combined with multiple other measures in a study such as ours it may lack ecological validity.^48^ This may explain why the results of our study did not replicate previous research.

While our findings suggest that sleep quality is associated with several risk factors underpinning smoking cessation, the results of our linear regression analyses also provide evidence of an association between fatigue severity and multiple factors associated with successful cessation and cessation beliefs. Furthermore, hierarchical regression analyses suggest that fatigue severity attenuates the associations between sleep quality and smoking urges and provides strong evidence that there is no longer an independent association between sleep quality and both barriers to cessation and risks of cessation when including fatigue severity in the model. This is important because although previous work has found subjective sleep quality is a strong predictor of subjective fatigue,^49^ our results provide evidence that fatigue uniquely explains variance in factors associated with successful cessation (eg, smoking urges) and beliefs (eg, barriers to and benefits of cessation). It is important to note that in unadjusted regression analyses variance of factors associated with successful cessation and cessation beliefs accounted for by fatigue severity was small, ranging from 11% (perceived risks) to 15% (barriers to cessation). Furthermore, we used a chronic measure of fatigue severity^34^ which asks fatigue-related questions about the previous week. Both acute sleep quality and fatigue may have a stronger association with smoking cessation variables and should be explored in future research.

These findings are consistent with past research identifying a relationship between fatigue, adverse health events, and behaviors.^25,50^ We found no smoking literature exploring the associations we observed in the present study; however, previous e-cigarette studies found fatigue severity predicted use behavior and multiple cessation beliefs.^26^ Our study provides evidence that participants with greater levels of fatigue may expect more problems when trying to quit despite recognizing that continued use is risky or harmful, both of which are associated with intentions to quit^51^ and actual treatment response.^52^ The mechanisms underlying this relationship are currently unknown and future work should endeavor to replicate our findings and identify plausible processes underpinning such associations. One potential area of future investigation is the link between fatigue and impaired emotion regulation^53^ and self-control.^54^ Both factors may play a role in this relationship, as individuals with higher levels of fatigue are less able to resist cravings to smoke or use emotion regulations strategies. Furthermore, fatigued smokers may use the stimulant effect of nicotine as a coping strategy which may result in a higher urge to smoker and worry more about the potential difficulties associated with quitting smoking (ie, loss of a coping strategy).

Our study has several limitations. First, because of our cross-sectional study design, we cannot determine the temporal direction of associations or infer causality. Future research should build on our findings by applying experimental or longitudinal designs to determine the nature of the relationship and causality. Second, although participants were recruited from 23 different countries, we did not collect ethnicity data, future work should employ recruitment strategies to ensure an ethnically diverse sample as there is evidence of racial disparities in sleep quality.^55^ Third, while our exposure and outcome measures are well-validated, interpretations should be considered with caution due to potential unmeasured confounding. Fatigue and sleep quality can be impacted by physical and mental health conditions, which may also impact outcome measures such as abstinence self-efficacy and/or cessation beliefs. This study did not account for physical or mental health conditions or medications, which may confound results and limit interpretation. Furthermore, we did not collect data on time of last cigarette, which may impact the interpretation of the QSU-Brief results, as smoking urges often depend on time of last cigarette and associated nicotine withdrawal. However, all participants in this study were smoking as usual (or were not asked to abstain). Therefore, we expect that levels of nicotine withdrawal would not be high or profoundly affect urges. Time since last cigarette would impact this measure, but participants with higher fatigue could display higher smoking urges for reasons unrelated to nicotine withdrawal. This could result in a residual association where higher fatigue severity is associated with higher smoking urges. Additionally, all measures were self-reported; therefore, smoking status was not determined objectively and consequently we may have a less accurate classification of smoking status.

Fourth, exposure variables (sleep quality and fatigue severity) were chronic measures, and future research should include acute measures which are likely to have better temporal contingency with outcomes associated with smoking cessation the day after a poor night’s sleep. Finally, our participants were recruited through the Prolific crowd-sourcing platform, which is likely to introduce some sampling bias. For example, individuals that do not have access to technology or the internet would have been unable to participate. Recruitment via Prolific may be affected by self-section bias; therefore, our sample may not be representative of the target population. In addition, Prolific predominantly uses convenience sampling, meaning that study places are filled on a first-come, first-serve basis. The platform identifies that it uses several mechanisms to reduce this bias, including processes to fairly distribute study places to active participants. However, the extent to which these actions mitigate bias is unknown. Therefore, the generalizability of our findings may be limited.

This is the first study to explore associations between sleep quality, fatigue severity, and smoking factors associated with successful cessation and cessation beliefs. Our data suggest that sleep quality and fatigue severity may be associated with multiple factors associated with successful cessation and cessation beliefs. Furthermore, our findings provide evidence that fatigue severity may have an independent effect on multiple factors associated with successful cessation when accounting for sleep quality. Further research is needed to extend these findings by using different methods to identify the temporal direction of associations and causality.

## Data Availability

Data are available in a public, open access repository. Study data, analysis code and associated documents are available at the University of Bristol data repository (https://doi.org/10.5523/bris.19aoqe6od86n72kigbdpzp89em)
